# Pain and Opioid Dependence: Is it a Matter of Concern

**DOI:** 10.4103/0973-1075.76240

**Published:** 2011-01

**Authors:** Agar Meera

**Affiliations:** Senior lecturer, Discipline of Palliative and Supportive Services, Flinders University, Daw Park, South Australia, Director of Palliative Care, Braeside Hospital, Hammond Care, Sydney, New South Wales, Conjoint Associate Professor, Sydney South West Clinical School, University of New South Wales, Sydney, Australia, Staff Specialist – Research, Sydney South West Area Palliative Care Service, Sydney, New South Wales, Australia

**Keywords:** Cancer pain, Opioid dependence, Substance abuse

## Abstract

Opioids are extremely effective in managing cancer pain, and now are utilized for longer periods of time in cancer patients as the treatment for malignancies has become more successful.[1] The goals in cancer pain treatment includes maintaining function in patients with cancer pain (especially in earlier stage disease), and palliation in advanced disease.[1] The perception of the lay public and inexperienced clinicians that addiction is inevitable, often leads to an inappropriate fear to utilize opioids to appropriately manage pain; resulting in persistent under-treatment of cancer pain internationally.[2,3] There is much confusion about the phenomenon of physical dependence and how this can be differentiated from the maladaptive behaviors that constitute a diagnosis of substance abuse. The burden of cancer and associated cancer pain is projected to continue to rise, and is often at an advanced stage at diagnosis in less developed countries.[4] To be able to provide quality care for this patient population availability of opioids and skilled clinicians in pain management is paramount. In the majority of cases, the main concern is to abate concerns about risks of opioid addiction; to allow adequate pain relief. To understand the infrequent phenomenon of substance abuse in the setting of cancer pain management clear definitions are needed, and review of the epidemiology of occurrence in cancer populations is needed. It is also important to clearly separate the issues of substance abuse at the patient level and diversion of prescribed opioids. There are principles of managing cancer pain in the rare clinical scenario when the risk of substance abuse is high, which can still allow safe management of cancer pain with opioids.

## DEFINITIONS

There is a significant variability in terminology utilized in research and clinical practice; and terms can lead to clinicians confusing pharmacological phenomena with abuse and addiction.[2] Maladaptive behaviors as defined by DSM IV–R need to be identified as being outside cultural and societal norms, also in the context of deterioration physical and psychosocial functioning related to progressive disease these changes can mimic core domains to identify “addiction”.[2] Equally a cancer patient who requests a specific opioid by a specific route may be demonstrating a good knowledge of their own cancer pain management and be assertive in the way they navigate health care systems.[2] The following are helpful definitions to use

### Tolerance

Tolerance is a “pharmacological property defined by the need for increasing doses to maintain the effect of the agent”.[2] In cancer pain management tolerance to some adverse effects may occur, but tolerance to analgesic effects requiring the need to escalate dose escalation is rare, and the need for dose escalation is usually in the context of a progressive painful lesion or new lesion developing.[2]

### Physical dependence

Physical dependence is defined as the occurrence of a withdrawal after abrupt dose reduction or an administration of an agonist.[2]

### Substance dependence and abuse

The DSM IV criteria for substance abuse and substance dependence provide definitions for maladaptive patterns of substance abuse, where the defining factor is compulsive drug seeking behavior.[1,2,5]

### Pseudo-addiction

The phenomena of “opioid seeking” may be seen when someone is experience unrelenting unrelieved pain and this is not be confused with substance dependence.[1,6]

## CLINICAL ISSUES RELATED TO PHYSICAL DEPENDENCE

The administration of an opioid antagonist or a mixed agonist–antagonist to person who has physical dependence will precipitate a withdrawal syndrome.[6] Opioid withdrawal is characterized by features including anxiety, nervousness, irritability, chills and hot flushes, salivation, lacrimation, rhinorrhoea, sneezing, sweating, gooseflesh, nausea and vomiting, abdominal cramps, and insomnia.[6] The time course of withdrawal is determined by the elimination half life of the opioid.[6] The avoidance of withdrawal has been proposed to support drug seeking behaviors, however, in cancer patients who pain is reduced or disappears due to effective anticancer therapies (e.g., radiation for painful bone metastasis) routine reduction or cessation of opioids occurs without significant issues.[2] It is proposed that reducing opioid doses to 75% of the previous daily dose and a down titration approach until dose is discontinued prevents withdrawal symptoms.[6]

## PREVALENCE OF SUBSTANCE ABUSE IN CANCER PATIENTS

There have been several studies exploring the prevalence of opioid addiction in medical illness. The most notable was the Boston Collaborative surveillance project, which only identified 4 cases of addiction out of 11,882 inpatients prescribed opioids.[7] The rates of substance abuse remain low and stable despite increased medical use of opioid analgesics, and this has also been replicated in study of 1723 outpatients and community care patients in an Indian context.[1,2,4]

Opioids used to manage cancer pain for patients with no prior history of substance abuse or addiction, is rarely associated with new onset of substance disorder.[2,7–9] The current understanding of the patho-physiology and etiology of substance abuse, supports the conclusion that the risk of *de novo* substance abuse or addiction when opioids are used for appropriate medical indications, is extremely low if no personal or family history or abuse or addiction or significant premorbid psychiatric disorder exists.

## PRINCIPLES OF CANCER PAIN MANAGEMENT WHEN PRIOR SUBSTANCE ABUSE OR RISK EXISTS

### Policy level

Policies need to be in place to allow secure storage of opioid medication stock, maintenance of records and stock registers and judicious prescribing by appropriately trained clinicians.[4] There are well-developed protocols internationally which meet these criteria stringently. Proper disposal of unused drugs and used opioid patches is also important. It is important that regulatory bodies and clinicians meet to discuss these issues so that the correct balance between drug control, and ensuring opioid availability for medical use occurs.[10]

### Clinician level

Clinicians need to be adequately trained in pain management, and the prescription and monitoring of opioid therapy; and have a responsibility to provide education to the patient and their family about their safe use, and adverse effect monitoring. A comprehensive history and assessment is needed prior to the commencement of opioid therapy, and attention to specific risks such as family history or significant premorbid psychiatric conditions.[1] Equally for the majority of patients, clinicians need to be comfortable allaying unfounded fears of opioid addiction, so that patients can receive adequate pain relief.

### Patient level

People with a history of abuse of opioids may develop cancer and associated pain syndromes; which in some cases may require opioid therapy for adequate relief.[11] In the at risk patient or a patient with known substance abuse, structure and a multidisciplinary approach is very useful, and it is important to realize/acknowledge they have significant cancer pain.[2] It is useful to have open and empathic communication with the patient about their drug use rather than avoid the topic. Assessment and treatment of comorbid psychiatric disorders is crucial for success of management in many patients. Opioids with slower onset or longer duration may be more appropriate choices; and the oral route is preferred.[1,6] The aim of therapy is usually harm reduction-minimizing harm from unrelieved pain balanced with harm due to substance abuse both to the patient and their surroundings.[6] Clear expectations in a written contract can be helpful for some patients and frequent monitoring with prescribing and dispensing by a single provider of a supply for a limited number of days (contingent on the patient coming for regular review). In the inpatient setting the clinical team need to be aware withdrawal symptoms may occur, and this may be related to more than one drug (and this includes alcohol and nicotine).

In the setting that an aberrant drug related behavior is identified, a comprehensive assessment and interpretation of these behaviors needs to be undertaken to determine the underlying cause, from a range of differential diagnoses.[2] The request for higher doses may be due to intractable pain, other underlying psychiatric diagnosis, which increase likelihood of the fear associated with a cancer diagnosis being manifested as impulsivity or aberrant drug taking, or self medication for coexisting symptoms such as depression, anxiety or insomnia. The patient may have a cognitive impairment or be confused about their medication regimen.
